# Impact of Electrospinning Parameters and Post-Treatment Method on Antibacterial and Antibiofilm Activity of Chitosan Nanofibers

**DOI:** 10.3390/molecules27103343

**Published:** 2022-05-23

**Authors:** Viktoriia Korniienko, Yevheniia Husak, Julia Radwan-Pragłowska, Viktoriia Holubnycha, Yevhen Samokhin, Anna Yanovska, Julia Varava, Kateryna Diedkova, Łukasz Janus, Maksym Pogorielov

**Affiliations:** 1 Biomedical Research Centre, Sumy State University, 2, Rymsky-Korsakov Str., 40007 Sumy, Ukraine; evgenia.husak@gmail.com (Y.H.); v.golubnichaya@med.sumdu.edu.ua (V.H.); justinsamokhin@gmail.com (Y.S.); yanovskaanna@gmail.com (A.Y.); yuliia.varava@gmail.com (J.V.); e.dedkova@student.sumdu.edu.ua (K.D.); 2 Faculty of Chemistry, Silesian University of Technology, 44-100 Gliwice, Poland; 3 Department of Biotechnology and Physical Chemistry, Faculty of Chemical Engineering and Technology, Cracow University of Technology, Warszawska 24 Street, 31-155 Cracow, Poland; lukasz.janus@doktorant.pk.edu.pl; 4 Institute of Atomic Physics and Spectroscopy, University of Latvia, Jelgavas iela 3, LV-1004 Riga, Latvia

**Keywords:** natural products, chitosan electrospinning, antibacterial biomaterials

## Abstract

Chitosan, a natural biopolymer, is an ideal candidate to prepare biomaterials capable of preventing microbial infections due to its antibacterial properties. Electrospinning is a versatile method ideally suited to process biopolymers with minimal impact on their physicochemical properties. However, fabrication parameters and post-processing routine can affect biological activity and, therefore, must be well adjusted. In this study, nanofibrous membranes were prepared using trifluoroacetic acid and dichloromethane and evaluated for physiochemical and antimicrobial properties. The use of such biomaterials as potential antibacterial agents was extensively studied in vitro using *Staphylococcus aureus* and *Escherichia coli* as test organisms. The antibacterial assay showed inhibition of bacterial growth and eradication of the planktonic cells of both *E. coli* and *S. aureus* in the liquid medium for up to 6 hrs. The quantitative assay showed a significant reduction in bacteria cell viability by nanofibers depending on the method of fabrication. The antibacterial properties of these biomaterials can be attributed to the structural modifications provided by co-solvent formulation and application of post-treatment procedure. Consequently, the proposed antimicrobial surface modification method is a promising technique to prepare biomaterials designed to induce antimicrobial resistance via antiadhesive capability and the biocide-releasing mechanism.

## 1. Introduction

Currently, antibiotic resistance is among the major global health problems and constitutes a common ground for biofilm-linked persistent infections. Biofilm formation is encouraged via bacterial communication, making biofilms more resistant to antimicrobial agents than bacteria growing in a free-swimming (planktonic) state. Microorganisms growing in a biofilm develop resistance via physical, physiological, and adaptive tolerance mechanisms. Biofilms are found in device-associated infection sites (catheters, joint prostheses, and prosthetic heart valves) [1]. Non-device-associated chronic and recurrent infections, including otitis, endocarditis, and urinary tract infections, are also associated with biofilm formation [2], despite the progress in infection control [3]. Antibiofilm materials prevent bacterial growth via inhibiting bacterial adhesion (antiadhesive surfaces) [4], killing bacteria (contact bactericidal effect) [5], or delivering the bactericidal agents (biocide releasing) [6]. However, the application of these materials has limitations due to the short life of such biomolecules as bactericidal agents [5].

Among broad-spectrum organic materials with antimicrobial properties, chitosan and chitin-based materials are receiving considerable attention from various scientific groups [7]. Chitosan (Ch), a mucopolysaccharide, is among the most abundant biopolymers in nature and is present in the exoskeleton of crustaceans, mollusks, insects as well as the cells walls of fungal cells. Due to its biocompatibility, biodegradability, antimicrobial activity, non-toxicity, and versatile chemical and physical properties, chitosan has become among the most attractive biopolymers in biomedical research [8]. Ch coatings prevent the adhesion and biofilm development of *E. faecalis* [9] and retard the growth of *Pseudomonas aeruginosa* upon contact [10]. Antibacterial and antibiofilm activity of chitosan, depending on its molecular weight, has been confirmed on *Streptococcus mutans* and *Streptococcus sobrinus* [11].

It has been reported that the properties of Ch depend on the structural organization of the initial polymer solution [12] and the method of chitosan-based material fabrication [13]. It has also been reported that Ch fibrous scaffolds demonstrate higher effectiveness than films, sponges, or gels [14]. Recently, electrospinning has become among the most popular methods for producing nanofibers from various synthetic and natural polymers [15]. This method allows for the production of materials with diameters below 100 nm that mimic the native extracellular matrix and may promote cell function and tissue regeneration [16]. Electrospun chitosan nanomaterials combined with silver nanoparticles (AgNPs) and polyvinyl alcohol (PVA) provide large surface-area-to-volume ratios as well as superior antibacterial properties due to AgNPs, which can be easily modified through its hydroxylic groups of PVA, thus to overcome the weak mechanical properties of chitosan [17]. Antibacterial activity of chitosan fibers against *Escherichia coli*, *Staphylococcus aureus,* and *Candida albicans* is dictated by the molecular weight (Mw) and degree of deacetylation (DD) of Ch [18].

However, electrospinning of chitosan solution is a highly sophisticated process due to its high viscosity and presence of free amino groups that form a positively charged polyelectrolyte under acidic conditions. Increasing the acid concentration in chitosan solution can decrease the surface tension and thus facilitate the electrospinning process [19]. Many organic [20] and inorganic acids have been tested for dissolving chitosan [21]. Furthermore, the type of solvent used also influences the biological activity of chitosan formulations. In this regard, dichloromethane (DCM) and trifluoroacetic acid (TFA) were found to be the most suitable solvents for electrospun chitosan fiber production [22].

However, these solvents influence chitosan structure, decreasing the mechanical parameters of membranes and facilitating degradation in an aqueous environment [23]. Therefore, the biomedical application of chitosan electrospun membranes requires neutralization, and the application of ethanol/methanol, sodium hydroxide (NaOH), or sodium carbonate (Na_2_CO_3_) aqueous solutions can provide efficient neutralization [24], decrease water solubility and increase biocompatibility [25].

The adhesion of bacteria is a complex multistage process that leads to biofilm formation. Various physical forces and physicochemical interactions initially lead to a reversible and, later, irreversible microbial adhesion. Microbial biofilm formation can be prevented via a direct-contact antibacterial effect.

Superior antimicrobial properties can be achieved by incorporating biocidal agents such as metallic nanoparticles in the biopolymer. Otherwise, electrospun nanofibers loaded with metallic nanoparticles such as AgNPs can have a cytotoxic effect on mammal cells [26]. On the other hand, successful management of co-solvent composition and controlled structural conformation applied to the suitable post-treatment procedure is significantly required considering the importance of the initial interaction between bacterial cells and nanofibers [27,28].

Considering the shortcomings in Ch electrospun membrane production for effective antibacterial applications, this study was undertaken to evaluate the antibacterial and antibiofilm effectiveness of electrospun Ch-DMC/TFA membranes using different post-treatment strategies.

## 2. Results

### 2.1. Chemical Structure Study

**Figure 1:**
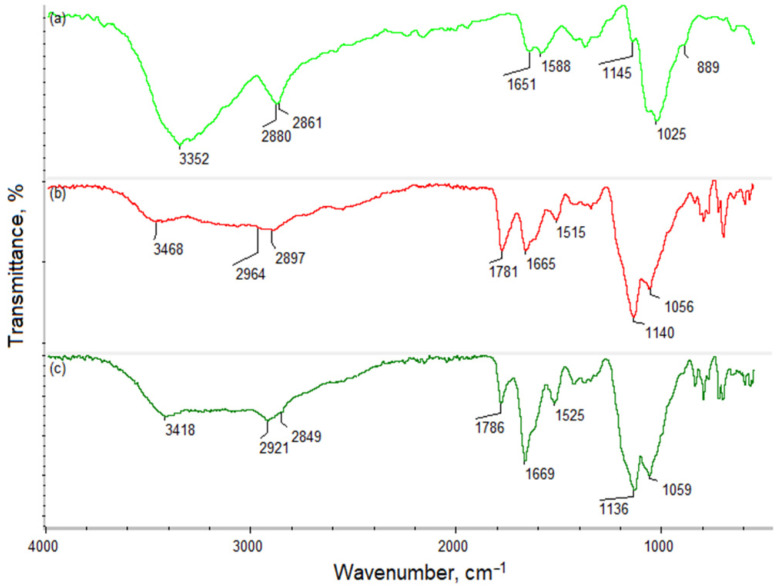
FT-IR spectrum of: (**a**) native chitosan; (**b**) chitosan treated with TFA/DCM 7:3 (sample 1); (**c**) TFA/DCM 9:1 (sample 2).

Chitosan owes its unique properties mainly to the NH2 groups of its polymeric chain. Figure 1 presents FT-IR analysis of the pristine polymer (a), Ch dissolved in TFA/DCM 7:3 (b), and Ch dissolved in TFA/DCM 9:1 (c). The spectrum of the native chitosan shows bands that are typical for free hydroxyl groups (3352 cm^−1^), aliphatic -CH_2_- and -CH_3_ moieties (2880 and 2861 cm^−1^), and amide bonds present in acetylated units (1651 cm^−1^). Additionally, bands coming from free NH_2_ are visible at 1588 and 1145 cm^−1^. Finally, there are bands at 1025 and 889 cm^−1^, typical for glycosidic bonds and glucopyranose rings. Figure 1b,c present the spectrum of chitosan after dissolving in TFA/DCM in different ratios (7:3; 9:1). The figure shows that there are some visible changes compared to the untreated Ch. There is a new band at 1781 cm^−1^, characteristic for free carboxyl and CF_3_ groups coming from trifluoroacetic acid. Noteworthy bands corresponding to amino groups are shifted to lower wave numbers (1515 and 1140 cm^−1^ for sample 1; 1525 and 1136 cm^−1^ for sample 2), which is typical for NH_3_^+^. Additionally, an increase in the intensity of the amide bonds can be noticed at 1665 cm^−1^ for sample 1 and at 1669 cm^−1^ for sample 2, which is a consequence of a chemical reaction between free carboxyl groups of trifluoracetic acid and free amino groups of chitosan. The change is especially significant in the case of TFA/DCM 9:1 application during Ch dissolution, due to the higher acid content. Bands corresponding to free -OH (3468 cm^−1^—sample 1; 3418 cm^−1^—sample 2), aliphatic groups (2964 and 2897 cm^−1^—sample 1; 2921 and 2849 cm^−1^—sample 2), and glycosidic bonds (1056 cm^−1^—sample 1; 1059 cm^−1^—sample 2) are visible as in the case of pristine chitosan.

### 2.2. Morphology of Nanofibers

**Figure 2:**
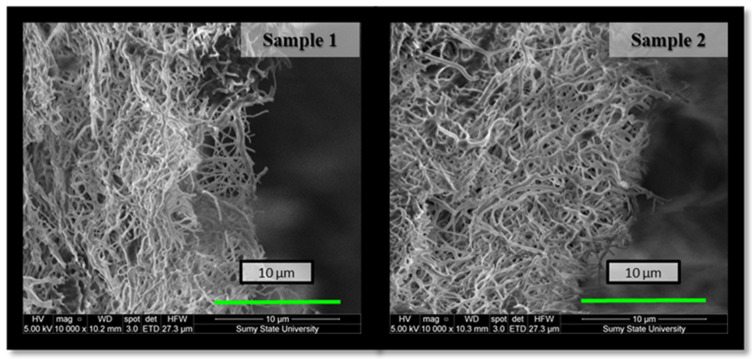
Cross-sectional SEM image of Ch-TFA:DCM 7:3 (sample 1) and Ch-TFA:DCM 9:1 (sample 2) as-spun membranes.

**Figure 3:**
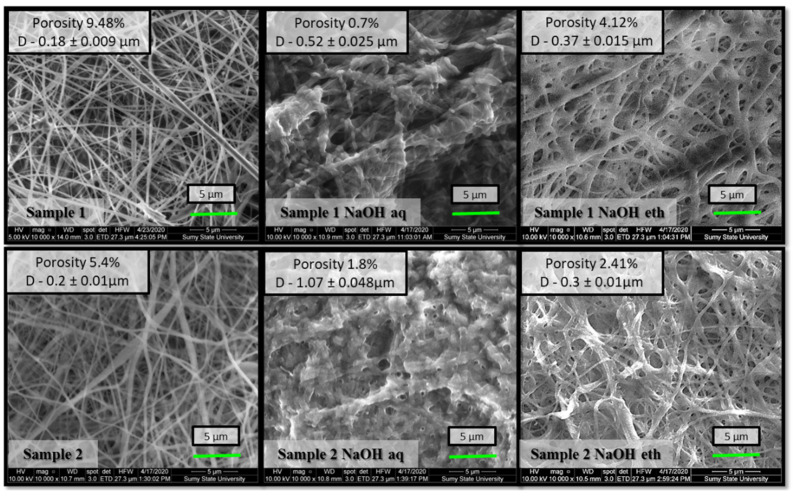
Scanning electron micrographs with ‘porous area fraction’ of sample 1 (Ch-TFA:DCM 7:3) and sample 2 (Ch-TFA:DCM 9:1) electrospun membranes after NaOH aqueous and NaOH ethanol treatment.

The choice of co-solvent is very critical for chitosan electrospinning to lower the surface tension of the solution and create a stable jet stream during the electrospinning procedure [29]. Fine, cylindrical, continuous, and randomly oriented Ch nanofibers were successfully prepared due to the high volatility of TFA and the possibility of blocking the positive charges of the amino groups on Ch. Such electrospun nanofibrous membranes were multilayered, porous, flexible, and permeable to liquid films with a high surface area. Their structure was determined by the cross-section method using a scanning electron microscope (Figure 2). SEM microphotographs also showed smooth fibers with diameters in the nano-range in both variants of membranes. However, the morphology of the deposited film depended on the TFA/DCM concentration in the solution. Although both sample 1 and sample 2 were fabricated under the same electrospinning conditions, their fiber diameter was 0.18 ± 0.009 and 0.2 ± 0.010 μm, respectively, and consequently deposited orientation significantly affected membrane porosity—‘porous area fraction’ (Figure 3) [30].

Figure 3 presents SEM results of as-spun nanofibers and after being treated with different alkali solutions. The samples of both co-solvent ratios displayed regular, randomly oriented, cylindrical, and bead-free nanofibers. After alkali treatment, the diameters of the nanofibers treated with 1 M NaOH aqueous solution increased slightly. The average diameter of non-treated sample 2 was measured as 0.2 ± 0.010 μm, enlarged to 1.07 ± 0.048 μm in the case of samples treated with 1 M NaOH aqueous solution, and only to 0.3 ± 0.01 μm with 1 M NaOH 70% ethanol. Our previous study also indicated that chitosan membrane coagulation with NaOH aqueous solution increased nanofiber size by swelling without changing fibrous morphology [31]. The ethanol solution provides gentler treatment and preserves the high porous structure better than the aqueous solution.

### 2.3. Antimicrobial Assessment In Vitro

#### 2.3.1. Antibacterial Efficiency and the Bacterial Reduction Rate

**Figure 4:**
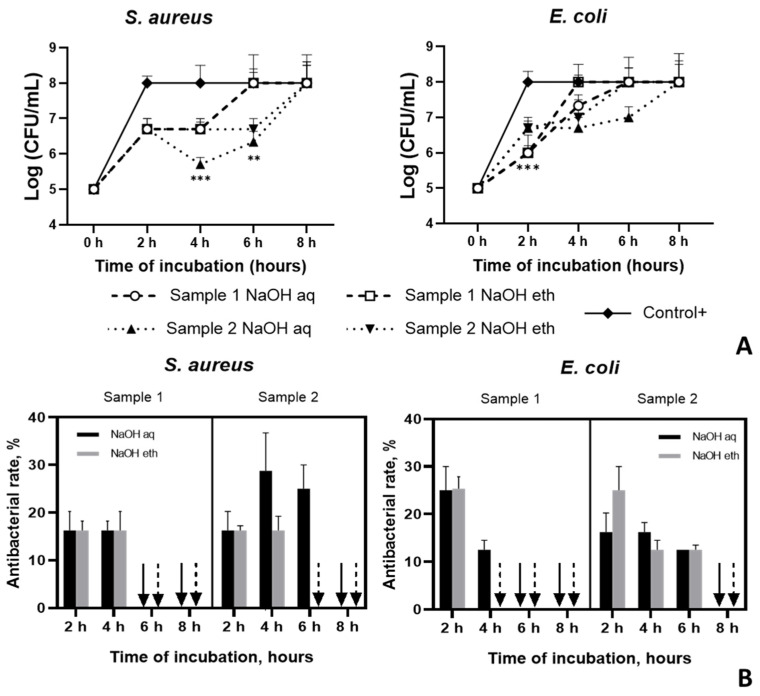
Antibacterial efficiency of chitosan membranes fabricated from TFA:DCM 7:3 and TFA:DCM 9:1 chitosan solutions against *S. aureus* and *E. coli*; (**A**) the number of bacterial cells, CFU/mL; (**B**) the reduction rate, %.

The antibacterial activities of Ch membranes were tested in vitro over an 8 h time period. Studies show that both Ch-TFA/DCM 7:3 (sample 1) and Ch-TFA/DCM 9:1 samples (sample 2) were more effective at inhibiting *E. coli* than *S. aureus* after 2 h incubation, whereas, after 4 h, *S. aureus* was more sensitive (Figure 4A). Furthermore, compared to Ch-TFA/DCM 7:3 membranes, Ch-TFA/DCM 9:1 specimens were more effective at inhibiting *E. coli* up to 6 h (*p* < 0.001). However, after 8 h, both samples exhibited no antibacterial activity against both *S. aureus* and *E. coli* (Figure 4B).

#### 2.3.2. Resazurin Metabolic Activity

**Figure 5:**
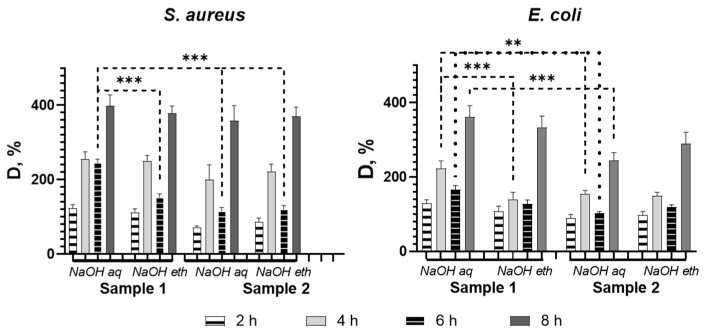
The capability of chitosan membranes to reduce biofilm formation based on resazurin reduction assay. Asterisks indicate p value between group: ** *p* < 0.01; *** *p* < 0.001.

Metabolic activity of biofilm cells was assessed by resazurin to determine the concentration of viable cells in the samples (Figure 5). In order to determine the correlation between the bacterial concentration in the sample and the intensity of the resorufin absorbance, we plotted a resazurin reduction chart for both bacterial species.

Both test organisms showed a similar trend in the survival rate of bacterial cells after incubation with the samples. Nevertheless, higher sensitivity value was observed for *E. coli* at all points of co-cultivation. All tested samples showed the highest bacteriostatic effect at 6 h. For both test organisms, D % values were still growing at the last incubation time of the assay (8 h) despite the solvent ratio and type of treatment. We found that biofilm eradication differed depending on the solvent system used for chitosan solution preparation. Studies show more effective bacterial biofilm reduction for Ch-TFA/DCM 9:1 (Sample 2). Overall, there were no significant differences in biofilm production between the two test organisms regardless of the treatment. Otherwise, *S. aureus* was less sensitive to sample 1 (Ch-TFA/DCM 7:3) treated with NaOH aqueous compared to all other tested samples (*p* < 0.001), while *E. coli* showed this tendency only in comparison to the similarly treated Ch-TFA/DCM 7:3 sample (*p* < 0.01), but at 4 h as well (*p* < 0.01).

#### 2.3.3. Morphology of the Bacteria Biofilm by SEM

**Figure 6:**
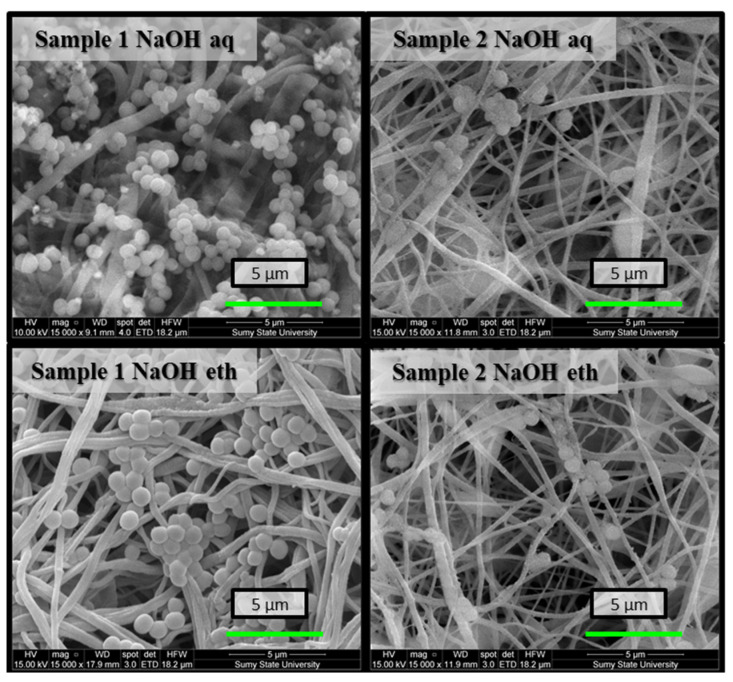
SEM images of bacterial biofilms on the surfaces of electrospun nanofibrous membranes after 8 h co-cultivation in the bacterial suspension of *S. aureus*.

**Figure 7:**
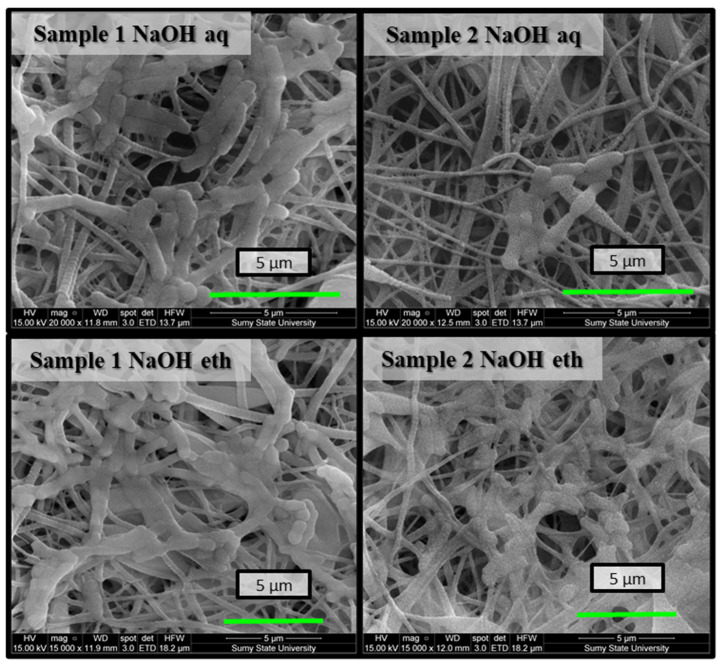
SEM images of bacterial biofilms on the surfaces of electrospun nanofibrous membranes after 8 h co-cultivation in the bacterial suspension of *E. coli*.

The results showed bacteriostatic performance by the Ch membranes on *E. coli* and *S. aureus*. Cell viability was lowest for both test organisms for Ch-TFA/DCM 9:1 (sample 2) membranes. As shown in Figure 6, there were a few bacterial colonies disseminated on the surfaces of the samples at the end of the assay. Otherwise, SEM observation indicated large numbers of *S. aureus* adhered on the surface of the Ch membranes forming a bead necklace structure. The cells of both organisms displayed membrane lesion or shape and cell size anomaly. The cell structure of *S. aureus* was destroyed, indicating the antibacterial performance of the nanofibrous membrane. Alteration of *E. coli* cell membrane integrity was also observed (membrane perforation led to the leaking of intracellular components). Subsequently, bacterial cells appear translucent and empty (Figure 7).

## 3. Discussion

Fiber morphology and average diameter, as well as membrane porosity, impact bacterial proliferation. Abrigo et al. have reported that bacteria favorably adhered to fibers with a diameter similar to bacterial size. The size of the pores in the electrospun Ch-TFA/DCM membranes was smaller than the size of the microorganisms, which prevented bacterial penetration and microbial growth. Further, bacterial shape also impacted adhesion—round *S. aureus* demonstrated higher adhesion and growth than rod-like *E. coli*. Fibrous networks constrain the colonization process, resulting in conformational changes to rod-shaped bacteria and inducing cell death [29]. Correspondingly, evaluation of antibacterial and antibiofilm activity verified the antibacterial resistance of electrospun chitosan membranes made of both solutions at the same level despite type of treatment. The FT-IR study showed that application of TFA/DCM solvents at different ratios affects the chemical structure of chitosan by increasing the number of protonated NH_3_^+^ groups, demonstrated by the higher intensity of the bands and their shift to lower wavelengths. Additionally, we have shown that the acid reacts with the polymer, creating covalent bonds (amide bonds), thus affecting the adhesiveness and morphology of the fiber. This effect can also be related to the incorporation of CF3 moieties revealed in the FT-IR spectrum. The change in the chemical structure was more visible for the sample prepared using a 9:1 ratio of TFA/DCM due to the higher amount of the reactive moieties of the acid (-COOH, CF_3_) and their higher availability. We revealed higher antiadhesive efficiency against *E. coli*, supported by SEM images.

Although Ch materials possess antibacterial potential, the properties depending on exposure time showed that co-solvent ratio limited antibacterial performance. Co-solvent ratio in a chitosan solution affects the antibacterial properties of the test samples on Gram-negative and Gram-positive bacteria in different ways: Ch-TFA/DCM 9:1 membranes showed superior antibacterial properties to Ch-TFA/DCM 7:3 films. Such phenomena can be explained by TFA forming chemical bonds with chitosan. Because of that, the polymeric chain gains fluorine atoms, which decrease Ch hydrophilicity, thus hampering bacterial adhesion.

**Figure 8:**
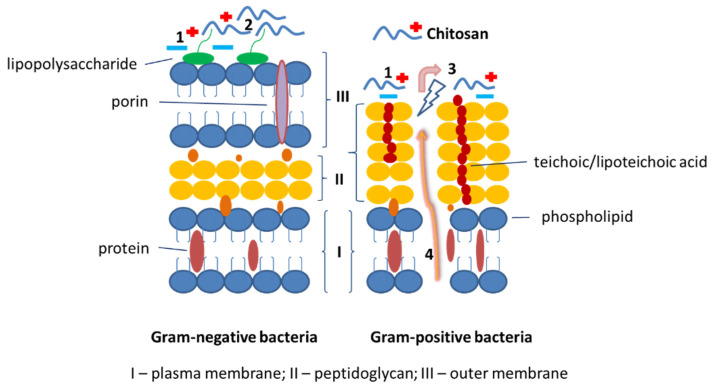
Schematic representation of chitosan mode of action on Gram-negative and Gram-positive bacteria cell wall. 1. Electrostatic adsorption and interaction between chitosan with cationic ions and bacteria cell wall. 2. The collapse of the outer membrane of the cell wall and blocking of nutrient flow. 3. Modification of cell permeability and disruption of the plasma membrane. 4. Osmotic damage and release of intracellular components.

Bacteria produce an extracellular substance as a survival mechanism to promote community-like transformation from the planktonic to the biofilm state. The biofilm state possesses resistance, which protects bacteria from antibiotic agents [32]. Antibiofilm molecules isolated from natural sources exhibit antimicrobial activity via neutralization and disassembly of lipopolysaccharides or alteration of membrane potential or membrane permeability [33]. NH_3_^+^ ions of chitosan interact with bacteria which are negatively charged (in Gram-positive bacteria due to the presence of teichoic acids and due lipopolysaccharides in the cell wall of Gram-negative bacteria) and thus inhibit their penetration [13,34]. Formulations containing a higher amount of TFA showed greater antimicrobial effects attributed to chitosan. This formulation reduced the microbial growth of both microorganisms at the early stage of incubation but was not effective after 8 h. This may be due to the time-dependent rate of chitosan degradation and oligomers released from nanofibers that allow implementation of the antimicrobial effect of chitosan through a shift in cell permeability, cell membrane destruction, and penetration of oligomers into the cell, leading to inhibition of transcription [14,35] (Figure 8).

## 4. Materials and Methods

### 4.1. Electrospinning

**Figure 9:**
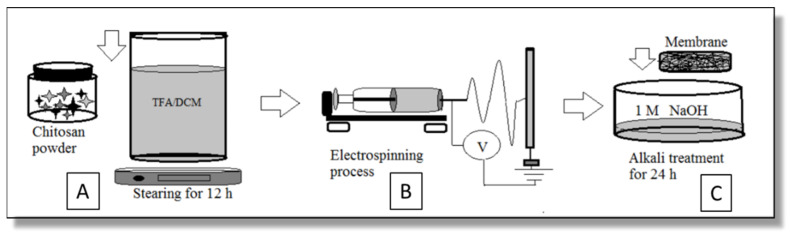
Schematics of sample preparation: (**A**) chitosan solution preparation; (**B**) electrospinning process; (**C**) alkali treatment of chitosan membrane.

Chitosan powder (MW 50–190 kDa, DD 75–85%, viscosity 20–300 cP) bought from Glentham Life Sciences (Corsham, UK) was dissolved in TFA/DCM (Sigma–Aldrich (St. Louis, MO, USA)) and blended to form two types of 3.5 wt% Ch solutions: Ch-TFA/DCM 7:3—sample 1 and Ch-TFA/DCM 9:1—sample 2, *v*/*v* (Figure 9A).

Electrospun membranes were made in an RT-advanced machine (Linari Engineering, Pisa, Italy) with the following parameters: electric field 30–35 kV, pump rate 5.0 mL/h, the distance between the needle tip and collector 15 cm (Figure 9B).

### 4.2. Neutralization

**Figure 10:**
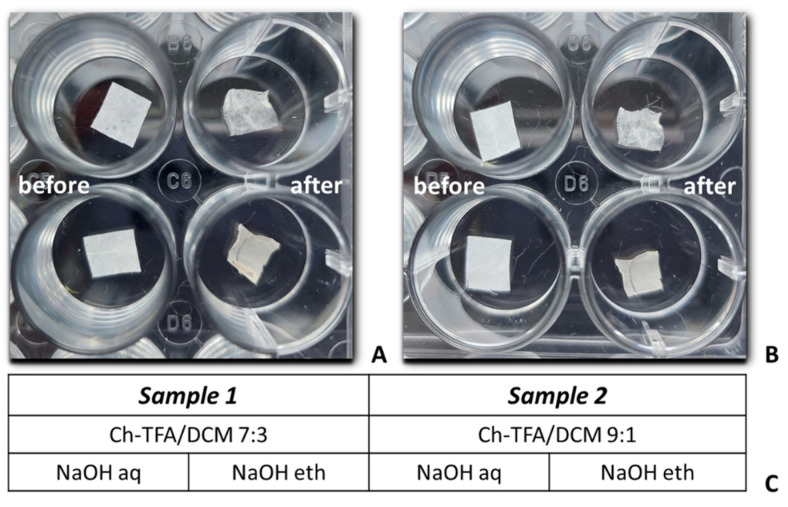
Macroscopic image of chitosan membranes—sample 1 (**A**) and sample 2; (**B**) before and after 24 h alkali treatment with 1 M NaOH aqueous (upper row) and 1 M NaOH ethanol solutions (lower row). Types of samples prepared for research (**C**).

NaOH (CAS 1310-73-2)) was purchased from Sigma–Aldrich (St. Louis, MO, USA). After electrospinning, membranes were placed in the wells of 24-well plastic plates and neutralized in 2 mL of 1 M NaOH solution, aqueous or 70% ethanol solutions for 24 h followed by washing three times in distilled water for 30 s and drying under ambient conditions for 24 h (Figure 9C). The macroscopic feature of the electrospun membrane before and after neutralization and the types of the samples prepared for investigation are shown in Figure 10.

### 4.3. Fourier Transform Infrared Spectroscopy (FT-IR) Analysis

FT-IR spectra were collected using a Thermo Nicolet Nexus 470 FT-IR spectrometer (Thermo Fisher Scientific, Waltham, MA, USA) in the range between 4000 and 500 cm^−1^. FT-IR was applied to analyze the chemical structure of the native chitosan and the biopolymer after processing. For the analysis, 50 mg of the dry sample was used. Each time, an Attenuated Total Reflection (ATR) diamond adapter was applied.

### 4.4. Scanning Electron Microscopy (SEM)

A scanning electron microscope (FEI, Brno, Czech Republic) was used to evaluate the inner structure of the membrane of the as-spun and post-treatment fibers. For better conductivity and reduced electron-charging effects, samples were observed as collected on aluminum foil (without any metallic coating) [23]. SEM high-resolution images were used to evaluate the spinnability and the presence of beads. The average fiber diameter, fiber diameter distribution, and porosity—‘porous area fraction’—were analyzed using image analysis software (ImageJ 1.518j, (NIH, Bethesda, MD, USA). The ‘porous area fraction’ of the fiber membrane was obtained by determining the area of the pores divided by the total area of the investigated image region. The ‘porous area fraction’ corresponds directly to a ‘local porosity’ [36]. All parameters were obtained from five SEM images for 100 fibers for each type of the investigated membrane.

### 4.5. Evaluation of Antibacterial Activity In Vitro

**Figure 11:**
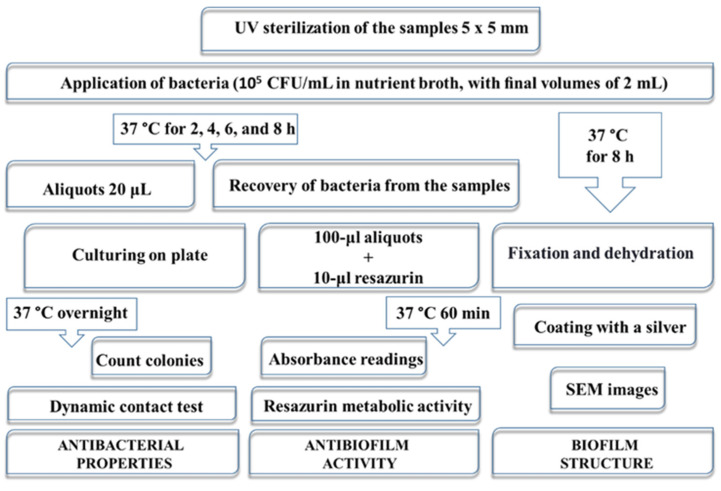
Schematic overview of the in vitro antibacterial tests.

The antibacterial properties were studied using a Gram-positive bacterium, namely, *Staphylococcus aureus* (*S. aureus*, B 918) and a Gram-negative bacterium, *Escherichia coli* (*E. coli*, B 926), obtained from the Bacteria Collection of Sumy State University by the National Collection of Microorganisms (Institute of Microbiology and Virology NASU, Kyiv, Ukraine). Cultures were incubated at 37 °C for 24 h. Then, bacterial suspensions were prepared and adjusted to an initial concentration of 10^5^ colony-forming units per milliliter (CFU/mL) with a liquid microbial growth medium. The antimicrobial performance of the Ch fibers was evaluated according to Figure 11. The dynamic contact test was applied for antibacterial efficiency and, consequently, bacterial reduction in vitro estimation. Ability to inhibit biofilm formation was assessed with a resazurin-based assay grounded on biological peculiarities of bacterial community behavior. SEM of the samples after 8 h incubation in bacterial suspension was carried out for biofilm bacterial structure assessment. Samples of 0.25 cm^2^ were placed in the laminar box (Porsa, Ukraine), and UV-light radiation at 254 nm was applied for 30 min on each side. The independent tests for samples and controls were completed in triplicates for both bacterial species. The experiments were carried out at a constant ambient temperature, humidity, and light.

#### 4.5.1. The Dynamic Contact Test

The dynamic contact test was used to determine the planktonic microbial population of a biofilm via calculation of log reduction in broth dilution assay. The samples were incubated in sterile flat-bottom polystyrene 24-well plates containing 10^5^ CFU/mL in nutrient broth, with final volumes of 2 mL for 2, 4, 6, and 8 h at 37 °C. At each time point, 20 μL of the medium was inoculated on agar plates using the spread plate method and incubated a 37 °C for 24 h. Afterward, the colonies were quantified as CFU/mL. The untreated bacterial suspension (10^5^ CFU/mL) was used as a control.

The reduction rate (R) of the total quantity of bacteria we calculated using the following Equation (1):

(1) $$R,\%=\frac{\left(C-E\right)}{C}\times 100,$$

where *C* and *E* are the amounts of surviving bacteria (CFU/mL) in the controls and experimental wells, respectively.

#### 4.5.2. Resazurin Metabolic Activity

The resazurin assay is based on the reduction of resazurin to resorufin by the bacteria. The resazurin method allows evaluating the cell growth within biofilms via detection of their metabolic activity. Towards this, after 2, 4, 6, and 8 h incubation at 37 °C, specimens were removed from experimental (the samples in bacterial suspension in an initial concentration of 10^5^ CFU/mL) and control (the samples in nutrient broth) wells, rinsed with phosphate-buffered saline (PBS) and transferred to 10 mL of PBS in 15 mL sterile disposable conical tubes, and then vortexed for 1 min at 1200 rpm. Subsequently, 100 μL aliquots from all experimental and control tubes were transferred to 96-well plastic plates (in three replicate wells). Afterward, resazurin was added in an amount equal to 10% of the control and experimental wells volume. Absorbances of microbial-generated resorufin were measured at 570 nm and 600 nm by Thermo Scientific Multiskan FC (Thermo Fisher Scientific, Waltham, MA, USA) microplate reader equipped with Skanlt Software 4.1 for Microplate Readers in 60 min periods of incubation at 37 °C. To calculate difference (D) % between experimental (E) and control (C) wells based on absorbance readings, the following formula provided by the manufacturer’s was used (2):

(2) $$Difference,\;\%=\frac{\left(\left(O2\times A1\right)-\left(O1\times A2\right)\right)\;}{\left(\left(O2\times P1\right)-\left(O1\times P2\right)\right)}\times 100$$

where *O*1— molar extinction coefficient of oxidized alamarBlue at 570 nm, *O*2— molar extinction coefficient of oxidized alamarBlue at 600 nm, *E*1—absorbance value of experimental wells at 570 nm, *E*2—absorbance value of experimental wells at 600 nm, *C*1—absorbance value of control wells at 570 nm, and *C*2—absorbance value of control wells at 600 nm.

#### 4.5.3. Morphology of the Bacteria Biofilm

After 8 h co-incubation of the electrospun membranes and bacterial strains (2 mL suspension with a concentration of 10^5^ CFU/mL), the samples were gently washed three times with PBS and fixed in 2.5% glutaraldehyde solution for 30 min twice. Afterward, the electrospun mats were washed with PBS, and gradually dehydrated in a growing concentration of ethanol (from 40% to 96%) for 30 min intervals for each concentration, followed by drying at room temperature overnight. The fixed samples were sputter-coated with a silver (VUP-5M, SELMI, Sumy, Ukraine) and imaged by SEM to investigate the bacteria’s morphological characteristics.

### 4.6. Statistical Analysis

Results were subjected to statistical analysis using the GraphPad Prism 9.1.1 software package. One-way analysis of variance (ANOVA) with three replicates was applied. *p* < 0.05 differences were considered to be statistically significant.

## 5. Conclusions

Electrospun Ch-TFA/DCM nanofibrous membranes provided a bacteriostatic effect on the planktonic bacteria and their biofilms. The reduction rate of bacteria was greater for nanofibers prepared using TFA/DCM 9:1 co-solvent system due to the incorporation of fluorine atoms into the chitosan polymeric chain backbone. Furthermore, the test results suggest that the morphological properties of electrospun fibers can control the formation of biofilm on the nanofibrous membranes. Despite NaOH alkali treatment leading to the growth of the diameter of the fibers by its swelling, it preserves the nanofibrous structure of the membrane. The test materials under investigation were able to inhibit the growth of both test microorganisms for up to 6 h, suggesting their higher antiadhesive potential and applicability for antibacterial applications. Nevertheless, both membrane compositions were more effective against *E. coli*, and NaOH aqueous post-treatment provided the most observable action. These studies encourage further investigations to use electrospun biofibrous materials as antibacterial agents to control bacterial adhesion and proliferation via structural and physicochemical features of the membranes, as well as the production of nanofibrous insoluble chitosan membranes suitable for biomedical application and tissue engineering.

## Data Availability

Data is available on request.
